# Orthodontic Extrusion vs. Surgical Extrusion to Rehabilitate Severely Damaged Teeth: A Literature Review

**DOI:** 10.3390/ijerph18189530

**Published:** 2021-09-10

**Authors:** Martina Cordaro, Edoardo Staderini, Ferruccio Torsello, Nicola Maria Grande, Matteo Turchi, Massimo Cordaro

**Affiliations:** 1 IRCCS, Fondazione Policlinico Universitario Agostino Gemelli, 00168 Roma, Italy; cordaromartina@gmail.com (M.C.); ferruccio.torsello@gmail.com (F.T.); nmgrande@gmail.com (N.M.G.); matteoturchi90@gmail.com (M.T.); massimo.cordaro@unicatt.it (M.C.); 2 Department of Orthodontics, Università Cattolica del Sacro Cuore, Largo Francesco Vito 1, 00168 Rome, Italy; 3 Department of Endodontics, Università Cattolica del Sacro Cuore, Largo Francesco Vito 1, 00168 Rome, Italy

**Keywords:** orthodontic extrusion, surgical extrusion, rapid orthodontic extrusion, forced orthodontic eruption, crown–root fracture, orthodontics

## Abstract

The need to rehabilitate severely compromised teeth is frequent in daily clinical practice. Tooth extraction and replacement with dental implant represents a common treatment choice. However, the survival rate for implants is inferior to teeth, even if severely damaged but properly treated. In order to reestablish a physiological supracrestal tissue attachment of damaged teeth and to arrange an efficient ferrule effect, three options can be considered: crown lengthening, orthodontic extrusion and surgical extrusion. Crown lengthening is considered an invasive technique that causes the removal of part of the bony support, while both orthodontic and surgical extrusion can avoid this inconvenience and can be used successfully in the treatment of severely damaged teeth. The aim of the present narrative review is to compare advantages, disadvantages, time of therapy required, contraindications and complications of both techniques.

## 1. Introduction

“Severely damaged teeth” are considered as teeth with severe structural damage due to multiple factors: crown–root fractures, extensive carious lesions, cervical root resorption or other causes that lead to the loss of part of the clinical crown. Such teeth need to be rehabilitated, even considering the high prevalence of subgingival root caries among the elderly [1].

Nowadays, in the so-called “dental implant era” (Clark and Levin), clinicians often choose to extract compromised teeth and replace them with dental implants, even if less invasive options are feasible [2]. Moreover, the lower implants survival rate compared to teeth should be considered even in cases of severely compromised but properly treated and maintained teeth [2]. Placing a dental implant is not free from possible intraoperative and postoperative complications, such as neurological damage and sinus penetration. No less, extraction is an irreversible action that should be considered as the last resort. 

A fixed partial prosthetic denture may represent another alternative to implant placement to replace extracted teeth, but it requires the inevitable mutilation of healthy dental tissue of the adjacent abutment teeth. The main limit of this choice is associated with the lower long-term survival rate than both dental implants and post-endodontically restored teeth [3].

For all these reasons, the maintenance and rehabilitation of a compromised tooth still seems to be the most effective treatment.

Due to prosthetic rehabilitation, it is crucial to create an effective ferrule effect for a desirable biomechanical behavior of the tooth [4]. The presence of 1.5–2 mm of sound supragingival dental tissue significantly increases tooth fracture resistance [4,5].

Even an adequate distance between crown margin and alveolar crest must be ensured to respect the supracrestal tissue attachment [4,6]. Supracrestal tissue attachment, commonly known as biological width, is defined as “the dimension of tissues placed coronally to the crest of the alveolar bone” [6,7]. Preserving it is a fundamental requirement for the health of periodontal tissues; any violation of its integrity can lead to gingival inflammation and consequent loss of clinical attachment and bone resorption [6].

Biological width is stated to be 2.04 mm, which represents the sum of epithelial and connective tissue average measurements with a significant intra- and inter-individual variability [6,7,8]. Therefore, the clinician should measure the individual dimension of biological width by performing a bone sounding, or ensure at least a distance of 3–4 mm between the alveolar crest and the crown margin [4,9,10].

In the case of a severely damaged tooth without sufficient supra-alveolar structure to achieve an effective ferrule effect and to ensure the preservation of the supracrestal tissue attachment, the clinician should assess as treatment options:

- Surgical crown lengthening;
- Orthodontic extrusion;
- Surgical extrusion [11].

Surgical crown lengthening, when performed for restorative purposes, requires the execution of an apically repositioned flap with bone resection [12]. As a general rule, it is necessary to remove an amount of bone in order to expose at least 4 mm of healthy dental tissue. The exposed healthy dental tissue will be then covered by gingival proliferation during healing from 2 to 3 mm [12].

The surgical technique requires the execution of an internal bevel incision whose distance from the gingival margin depends on the pocket depth and on the keratinized gingiva width. Two relief incisions can be added [12]. A full-thickness flap is performed to expose the bone; by using fissure burs and/or bone chisels, bone recontouring is carried out on the compromised tooth and extended to the adjacent teeth to harmonize the gingival morphology. Finally, the flap is repositioned at the level of the bone crest and sutured [12]. Bone removal can cause a significant worsening of the crown/root ratio, exposure of the furcation and severe weakening of the periodontal ligament of the tooth to be exposed and of the adjacent teeth [1,4,13]. Therefore, this technique is contraindicated in the exposure of subgingival lesions of a single tooth, especially in aesthetic areas [12].

More conservative techniques, such as orthodontic extrusion and surgical extrusion, should be preferred to restore severely compromised teeth.

The aim of this review is to compare advantages, disadvantages, time of therapy required, contraindications and complications for both techniques.

## 2. Materials and Methods

In order to analyze both orthodontic extrusion and surgical extrusion, a literature search was performed using PubMed database. The keywords used were “orthodontic extrusion”, “forced orthodontic extrusion”, “forced orthodontic eruption” and “surgical extrusion”; no filter was applied regarding patients’ age, sex and country. Original articles and reviews in the English language describing one or both techniques were selected based on title and abstract. The reference list of the included articles was analyzed and added to the screening phase. No restrictions were placed on the publication status of the articles or year.

## 3. Results

A total of 57 articles on surgical extrusion, 52 articles on orthodontic extrusion, 4 articles on ferrule effect, 9 articles on biological width and 16 articles on surgical crown lengthening were selected. These were divided into groups (e.g., orthodontic extrusion group, surgical extrusion group, etc.) and completely read. After full-text screening, 12 articles were eligible for the qualitative summary of the results.

## 4. Discussion

Based on the literature review, orthodontic extrusion and surgical extrusion will be discussed by analyzing the advantages, disadvantages, indications, contraindications and complications of each technique. The results are summarized in Table 1. A combined therapy, that merges the advantages of both techniques, is proposed as a future therapy to rehabilitate severely damaged teeth.

### 4.1. Orthodontic Extrusion

Orthodontic extrusion, also known as forced orthodontic extrusion or forced orthodontic eruption, is defined as tooth movement caused by coronally directed orthodontic forces. It is performed to change tooth position or induce therapeutic changes on the surrounding alveolar bone and soft tissue [14].

It is indicated:

- In the treatment of traumatized or impacted teeth (canines) [14,15,16,17,18];
- To expose dental structure to facilitate tooth restoration in the case of a subgingival or infraosseous lesion or to confer an adequate ferrule effect, especially in aesthetic areas [14,15,19];
- To correct a biological width violation [15];
- To reduce angular bone defects [14,15,20,21,22];
- To correct an inadequate gingival zenith level and to modify the gingival architecture [14,23,24];
- For peri-implant purposes, to increase the volume of the alveolar ridge and the keratinized gingiva [15,23,25];
- To perform orthodontic extraction, when surgical extraction is contraindicated [15].

It is contraindicated in case of ankylosis or hypercementosis (since the extra load would cause the intrusion of the anchoring teeth), vertical root fracture, proximity to the roots of contiguous teeth, severe internal or external root resorption, short root length, untreated periodontitis or periapical disease and when it would cause the exposure of the furcation in multi-rooted teeth [14,15,26].

In 1973, Heithersay first described the use of orthodontic extrusion to move coronally the remaining root of fractured teeth [14,27].

**Figure 1:**
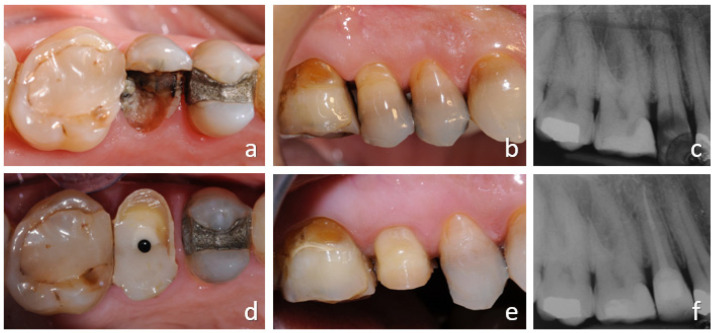
Orthodontic extrusion of tooth 1.5 with insufficient ferrule effect. Preoperative clinical occlusal and vestibular views and radiograph (**a**–**c**): tooth with deep caries. Endodontic treatment and post-endodontic restoration with post was performed: occlusal and buccal views and post-operative radiograph (**d**–**f**).

**Figure 2:**
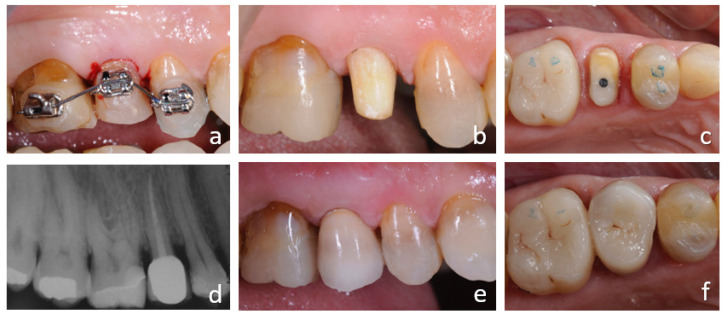
Orthodontic extrusion of 1.5 with insufficient ferrule effect. Orthodontic therapy to extrude tooth 1.5 (**a**). Tooth preparation after orthodontic extrusion: buccal and vestibular views (**b**,**c**). Prosthetic rehabilitation: post-operative radiograph and buccal and occlusal views (**d**–**f**).

Orthodontic extrusion (Figure 1 and Figure 2) exerts only tension on the surrounding tissues, and this kind of orthodontic force stimulates marginal bone apposition through increasing osteoblastic activity. Therefore, orthodontic extrusion can be used to correct intraosseous defects [14,28]. Similarly, it is possible to modify soft tissue morphology with an increase in the amount of keratinized gingiva, identifiable at first as a “red patch” [15,27]. Based on this concept, in 2018 “Guided orthodontic regeneration” was proposed by Paolone et al., to ensure the reconstitution of dental support tissues by using extrusive orthodontic movements [23].

Hard tissue increment is advantageous if orthodontic extrusion is carried out to correct infraosseous defects or for pre-implant purposes, since the increased keratinized tissue can improve the aesthetic result of the final prosthetic restoration [27]. On the other hand, it may show an undesirable effect if extrusion is performed to expose the subgingival structure of a tooth to be restored. This would require a periodontal surgery to expose subgingival lesions or healthy dental tissue needed for restorative purposes.

To prevent gingival coronal migration, the use of circumferential fiberotomy, namely the 360° incision of the supracrestal gingival fibers, has been proposed [15,27]. It is usually performed with a sharp scalpel blade inserted in the sulcus and used circumferentially to sever the fibers. A small injection of anesthetic is required.

Especially when combined with root planning, the fiberotomy was more effective in preventing coronal soft tissue migration than orthodontic extrusion alone [29,30]. Root planning takes away the supracrestal gingival fibers remaining on the tooth surface and previously cut by the fiberotomy. The recovery of the previous attachment level hinged on the reinsertion of the supracrestal gingival fibers; the fiberotomy prevents their reinsertion, thus obtaining the optimal crown length to be restored [30].

However, there is a broad disagreement in the literature about the correct timing to perform this technique. Some authors recommend a weekly fiberotomy [15,31,32]. Others suggest to perform fiberotomy only once, when the orthodontic movement is completed, just before the surrounding tissue remodeling occurs, or immediately before extrusion, so that the tooth can extrude without dragging the gingiva with it [15,33,34].

Regarding the timing of fiberotomy, many clinicians consider this technique unpredictable, as a corrective periodontal surgery at the end of the treatment may be still required to correct tissue level discrepancy between the tooth extruded and adjacent teeth [15].

Orthodontic extrusion can be obtained using different orthodontic strategies: fixed appliances, removable appliances and temporary anchorage devices such as mini-screws [11,35]. Even the use of neodymium–iron–boron magnets has been proposed [36]. Different treatment choice variables must be considered, such as patient preference, oral hygiene, availability of an appropriate orthodontic anchorage and amount of dental crown available [11]. 

In the case illustrated in Figure 1 and Figure 2, a severely damaged upper second premolar was restored. The tooth presented a long root (Figure 1c), which was considered a favorable condition for tooth extrusion. The root canal treatment and a provisional restoration with carbon-fiber post was performed (Figure 1d). The crown height was reduced to allow the extrusion of the tooth without interference with the opposing arch and to avoid tooth fractures which are frequent in the endodontically treated teeth.

Three braces were bonded on teeth 1.6, 1.5 and 1.4, taking care to position the bracket on tooth 1.5 approximately 3 mm more apically than the neighboring teeth.

Intrasulcular fiberotomy was performed with a 15c surgical blade 360° around the second premolar. Scaling and root planning of this tooth was also carried out to contrast the tendency of fibers to re-attach to the tooth.

A sectional 0.014 NiTi wire was tied to the three brackets, thus developing an extrusive force on tooth 1.5 and counteraction forces on teeth 1.4 and 1.6. A stainless-steel round 0.020 wire was bonded on the palatal surfaces of teeth 1.4 and 1.6 so as to splint them to create an anchorage unit.

After the successful extrusion, the tooth was prepared with a knife edge finishing line (Figure 2c) for a provisional resin crown and finally restored with a monolithic zirconia crown (Figure 2d–f). 

#### 4.1.1. Advantages

Orthodontic extrusion is a safe, minimally invasive and highly predictable treatment, rarely associated with complications [14,15,34,37,38].

It shows several advantages if compared to surgical crown lengthening. First, tooth structure and periodontal support maintenance is crucial: as previously mentioned, in surgical crown lengthening it is required to extend bone resection on the adjacent teeth to harmonize the gingival morphology. This causes bone loss and a possible damage of the periodontal support as well as a worsening of the crown/root ratio of these teeth [15]. Conversely, orthodontic extrusion can just cause tooth displacement or even increase the volume of dental support tissues, which is particularly advantageous for implant purposes [23].

The combination of orthodontic extrusion and fiberotomy can be particularly advantageous in highly aesthetically demanding areas, where surgical crown lengthening on a single tooth could lead to an unsatisfactory aesthetic result [14,30].

Finally, a relatively simple tooth movement is required to perform orthodontic extrusion: it is considered the easiest among the orthodontic movements as it simulates the physiological dental eruption [15,39]. 

#### 4.1.2. Disadvantages

The main disadvantage of orthodontic extrusion is treatment time, with an average of 4 to 6 weeks [15]. In addition, from 4 weeks up to 6 months of retention may be required, depending on the treatment goal [15]. For this reason, clinicians and patients may not choose orthodontic extrusion as the first treatment option [15].

Furthermore, if fiberotomy is performed weekly, a high patient compliance is requested with the possible need of periodontal surgery at the end of the treatment [15]. 

As all orthodontic devices, it can cause oral hygiene worsening and aesthetic problems [15].

#### 4.1.3. Complications

Orthodontic movements, especially if performed on traumatized teeth or through the application of heavy forces, can cause root resorption [14]. However, it can be considered a rare event when performing extrusive movement [14,27,34].

If heavy forces are applied, the risk of causing intrusion of the anchoring teeth and ankylosis of the tooth to be extruded increases, due to the trauma exerted on the periodontal ligament [15,27,40,41]. 

Relapse, namely the intrusive dislocation of the extruded tooth, represents an undesirable event much more frequent than the previous ones.

Fiberotomy, regardless of whether it is performed before or after orthodontic movement, and a retention period of at least 3–4 weeks, help to reduce this undesirable effect [14,27,30,34,42,43,44].

### 4.2. Surgical Extrusion

**Figure 3:**
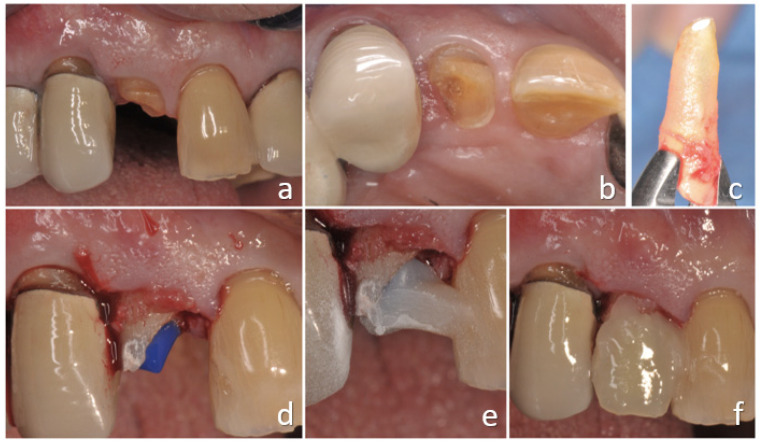
Surgical extrusion of tooth 1.2 with insufficient ferrule effect (**a**,**b**). Tooth was extracted with an atraumatic method (**c**) and then reimplanted and splinted, waiting for healing (**d**–**f**).

**Figure 4:**
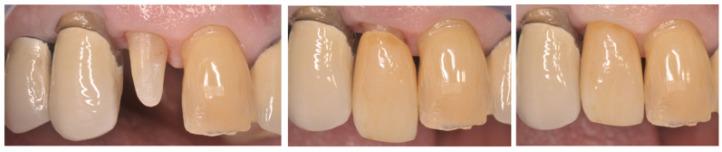
Surgical extrusion of 1.2 tooth with insufficient ferrule effect. Prosthetic rehabilitation.

**Figure 5:**
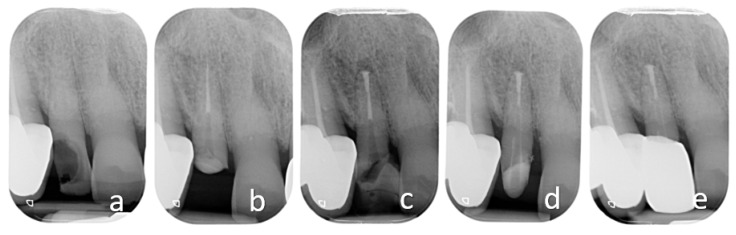
Surgical extrusion of 1.2 tooth with insufficient ferrule effect. Preoperative radiograph (**a**). Primary endodontic treatment (**b**). Reimplantation after retrograde endodontic treatment and splint (**c**). Tooth preparation and prosthetic rehabilitation after healing process (**d**,**e**).

Surgical extrusion (Figure 3, Figure 4 and Figure 5), also known as intra-alveolar transplantation, is the procedure whereby the remaining structure of a tooth is intentionally replaced to a more coronal/supragingival position in the same tooth’s original socket [45,46,47].

This technique is advocated as an alternative to extraction, orthodontic extrusion and surgical crown lengthening for the rehabilitation of severely compromised teeth. It allows the relocation of a subgingival lesion or the margin of a tooth more coronally in order to restore it, conferring a sufficient ferrule and respecting the biological width space [11,45,46,48].

Surgical extrusion can be performed with or without the complete tooth removal from the alveolus: the tooth can just be moved coronally or it can be extracted from its alveolus and re-implanted more coronally.

Since there are no significant differences in success rate between surgical extrusion without tooth extraction and surgical extrusion associated with extraoral manipulation and intentional replantation, tooth extraction is convenient in the majority of cases. Indeed, this allows the clinician to visually inspect the root surface and the anatomical structures of the apical area, with a single apical foramen or multiple portals of exit. This procedure should be performed under magnification with loupes; or better, with an operative microscope. In many cases, it is necessary to endodontically treat the tooth with an apicectomy, a retrograde preparation and cleaning of the root canal and the sealing of the preparation. The root resection is made using high-speed burs and it is performed following the same indications used in apical surgery (resection of 3 mm of apical root length); the resection can be shorter when a pathological root resorption is present or when the overall length of the root is not compatible with such resection length. The retrograde preparation can be performed using ultrasonic surgical retrotips or small high-speed rotary burs used in a delicate way, respecting the axis of the root canal; the retrograde sealing is performed using putty such as bioceramic materials condensed using the same instruments used in apical surgical procedures [45,49,50,51].

The extraoral inspection of the root surface enables the identification of anatomical variations, secondary canals and cracks spread along all surfaces of the root [45]. Indeed, it has been shown that in approximately 30% of teeth with crown–root fractures, replantation was not possible due to severe cracks or fractures already present on the root surface [52].

By intentionally replanting the tooth to extrude, it is possible to manage complex endodontic cases that are difficult to treat with conventional orthograde or retrograde techniques, such as natural or iatrogenic root canal obstruction and proximity to important anatomical structures, or in cases where root damage is not easily accessible or repairable intraorally, such as cervical root resorption [45,53].

These indications were described by Grossmann in 1996 for intentional replantation. Even if intentional replantation differs from surgical extrusion because the tooth is repositioned at the same level where it was originally, indications for the latter are the same [45,54,55].

Both intentional replantation and surgical extrusion are indicated in permanent teeth with an ideal root anatomy to perform an atraumatic extraction [45]. This condition is often satisfied in single-rooted teeth. In multi-rooted teeth with unfavorable anatomy, the attempted extraction can result in severe damage of the root surface or tooth fracture [45]. Therefore, in this type of tooth, surgical extrusion and intentional replantation are contraindicated [45]. A preoperative CBCT may be indicated, especially in multi-rooted teeth, to assess the suitable root morphology [45,56,57,58].

Despite the severe advantages mentioned above, surgical extrusion and intentional replantation are often considered by many clinicians as a last-resort procedure [45]. This is supposedly due to the common concern to inevitably cause damage to the periodontal ligament during extraction and the consequent risk to develop ankylosis and root resorption [45].

The clinical procedures of both surgical extrusion and intentional replantation start with local anesthesia and incision of the supracrestal fibers of gingival attachment. In various studies, a systemic antibiotic prophylaxis is suggested [59,60,61,62,63,64,65]. In order to limit any potential root surface damage, luxation should be avoided or performed with particular care, and only rotatory movements should be preferred. After tooth extraction, it should be visually inspected, preferably under magnification, to check for root fractures or severe cracks that would compromise treatment success (Figure 3c). During extraoral manipulation, the tooth should be firmly held, to avoid periodontal ligament damage, and it should be irrigated with sterile saline solution [50]. If required, apicectomy and retrograde sealing can be performed as previously described [66]. The extraoral handling time should not exceed 15 min; if more time is needed, the survival rate could be lowered [45,51,67]. Once the seal has been performed and the blood clot removed, it is possible to replant the tooth in its socket to the level that is clinically convenient [45]. It is possible to rotate by 90° or 180° the root before reinserting it to facilitate the exposure of the lesion or improve the ferrule [68]. After the reimplantation, it is suggested to verify the level of repositioning and carefully check the occlusal contacts to adapt them in case of precontact; at the same time, the overall position of the tooth should be evaluated with a radiograph. Once the tooth is replanted, it should be splinted using a semirigid orthodontic splint bonded to the adjacent teeth; this kind of splint permits post-op minimal adjustments of the position and it is recommended when compared with rigid splints [45,69,70]. The time of splinting retention depends on tooth post-operatory stability, though it usually ranges between 1 and 3 weeks [71,72].

Success criteria include periodontal healing without signs of progressive root resorption or ankylosis, the absence of significant marginal bone loss and the absence of tooth mobility beyond physiological limits [45,46].

Variables influencing the outcome are represented by an atraumatic extraction method, which provides the least possible damage to the cementoblast layer on the root surface, and by a rapid extra-alveolar handling, lasting less than 15 minutes, without using chemicals potentially harmful to periodontal ligament cells’ survival, such as sodium hypochlorite [73]. 

#### 4.2.1. Advantages

A short time to extrude the tooth is beneficial; in a single session, the desired amount of extrusion can be obtained.

It is possible to endodontically treat the tooth simultaneously with extrusion; apicoectomy and retrograde filling can be performed outside the alveolus, visually checking the seal.

Furthermore, the achievement of aesthetic results and the low incidence of failure documented so far in the literature should encourage its use [46,47,48,74,75,76,77,78,79,80,81].

Finally, surgical extrusion compared to surgical crown lengthening allows a better maintenance of the interproximal papilla and less marginal bone loss [81].

#### 4.2.2. Disadvantages

As mentioned above, both surgical extrusion and intentional replantation are not indicated for teeth with root morphology non-compatible with atraumatic extrusion. Moreover, the risk of causing root resorption or ankylosis due to a non-cautious handling of the periodontal ligament, as well as the wide discrepancy in success rate of the results reported by various studies and the absence of a universally established protocol, do not encourage clinicians to choose this therapeutic option [45].

#### 4.2.3. Complications

The most frequent complication associated with this technique is non-progressive root resorption, which can affect up to 30% of cases [11,71].

Other complications are tooth fracture during extraction, progressive root resorption, marginal bone loss and persistent mobility that can lead to tooth loss [11,71].

### 4.3. Combined Therapy

As previously described, intentional replantation can be pursued if traditional orthograde and/or retrograde endodontic techniques have failed or are considered unfeasible [45,82].

One of the factors that most influences the success of this procedure is the preservation of periodontal ligament of extracted tooth. Preventing chemical and mechanical damage through a rapid and careful extraoral tooth manipulation and the execution of extraction as atraumatically as possible can improve the prognosis [45,83].

Extraction movements, therefore, are closely related to the success of intentional replantation and to the development of complications, such as ankylosis and root resorption, associated with this technique [45,53,82,83].

For this reason, various atraumatic extraction methods have been proposed for both intentional replantation and surgical extrusion, such as vertical extraction procedures [84]. The most recommended extraction method is still holding the tooth with forceps beyond the CEJ, using preferably rotary movements [45,83].

It is important to consider that most teeth requiring intentional replantation have a weakened dentinal structure due to previous root canal treatments or retreatments, extensive restorations and root canal posts [82,83]. Therefore, a firm grasp during tooth extraction can lead to their fracture [83].

To overcome this problem, in 2010, a new protocol called “Atraumatic Safe Extraction” (ASE) was introduced by Choi et al., whereby a preoperative orthodontic extrusion for 2–3 weeks is performed before surgery to increase the mobility and volume of the periodontal ligament of the tooth to be intentionally replanted [1,82,83]. The preoperative orthodontic mobilization makes extraction easier, thus reducing the risk of complications such as root resorption and ankylosis [82,83].

The same authors, in 2014, in a comparative retrospective study between intentional replantation with and without ASE technique, found that preoperative orthodontic extrusion reduces the risk of root resorption and tooth fracture during extraction, thus statistically significantly increasing the survival rate of the intentionally replanted teeth [83]. Other variables, such as age, sex, tooth type and position, do not seem to influence its survival [83].

Preoperative orthodontic extrusion can be useful, especially if intentional replantation is planned on teeth with complex root anatomy, a condition associated with a higher risk of fracture during extraction, or if the tooth to be replanted has received repeated non-surgical root canal treatments that have weakened roots or in the case of a tooth with a small residual coronal structure [82,83].

A similar approach, termed “ortho-transplantation”, was previously proposed by Hayashi to prevent postoperative root resorption in tooth autotransplantation [85]. The author suggested that performing a preoperative orthodontic extrusion would increase the volume of the periodontal ligament of the donor tooth, preventing root resorption after transplantation [83,85]. In the ASE protocol, the same concept was adapted as that of intentional replantation to prevent root resorption [1,83]. Similarly, the same methodology should be applied to surgical extrusion techniques as well; its use has been described only in a retrospective study by Choi et al. in 2019 [1]. In this method, orthodontic forces of about 50 g are applied using a system of orthodontic brackets or orthodontic brackets and buttons and a Ni-Ti arch 0.014 [1,82,83]. In the posterior teeth, a 0.016 × 0.022 arch can be bent as a L to achieve extrusion [83]. 

Once tooth appears 1–2 mm extruded and its mobility increases, the extraction is carried out using Physics forceps, extractive forceps developed by Misch and Perez in order to minimize damage to the root surface during extraction. It is possible to proceed further with the classic intentional replanting protocol [1,82,83]. The extracted tooth is kept under constant hydration with cold physiologic saline, visually inspected and then reimplanted more coronally, as much as needed to expose the subgingival lesion, though considering the crown/root ratio.

## 5. Conclusions

Both surgical and orthodontic extrusion can be used successfully in the treatment of severely compromised teeth.

It is advisable to perform surgical extrusion if there is the necessity to solve endodontic problems that cannot be treated with conventional orthograde endodontic techniques, or as an alternative to the extraction of teeth that cannot be alternatively restored.

It is preferable to choose orthodontic extrusion if a highly predictable treatment is requested, if an orthodontic device is already present and if it is necessary to preserve tooth vitality or treat teeth non-compatible with an atraumatic extraction. 

A future perspective to pursue is a combined technique, able to merge the advantages of each technique.

By performing a preliminary orthodontic mobilization, a procedure that increases the volume of the periodontal ligament, it is possible to make the extraction much less traumatic while performing surgical extrusion. This approach drastically decreases tooth fracture risk, which is the most frequent cause of failure related to surgical extrusion. Compared to orthodontic extrusion, the time and patient compliance required are considerably reduced: on average, only 2–3 weeks of orthodontic mobilization are sufficient to proceed with surgical extrusion. Therefore, there is no need to perform fiberotomy, which may cause considerable discomfort, especially if it is performed weekly. 

Further studies on orthodontic extrusion preliminary to a surgical extrusion are needed.

## Figures and Tables

**Table 1:** Indications, contraindications, advantages and disadvantages of both orthodontic extrusion and surgical extrusion.

	Orthodontic Extrusion	Surgical Extrusion
Indication	Rehabilitation of teeth with subgingival lesions or that are severely damaged; Treatment of restorations that violate the biological width; Correction of angular defects and pink aesthetic; Regeneration of the alveolar ridge (for implant purposes); Orthodontic extraction; Treatment of impacted teeth (canines).	Teeth that cannot be restored with conventional techniques with root anatomy compatible with atraumatic extraction (single-rooted teeth). Teeth with endodontic clinical scenarios difficult to treat through classic procedures that need to be extruded.
Contraindications	Ankylosis or hypercementosis; Vertical root fracture; Close proximity to the roots of adjacent teeth;Severe internal or external root resorption; Untreated periodontitis or periapical disease; Short roots and exposition of furcation in multirooted teeth.	Teeth with root anatomy not compatible with atraumatic extraction (e.g. multi-rooted teeth with divergent roots). Medical contraindications to any surgical therapy.
Advantages	Minimally invasive treatment: no loss of bone or periodontal tissue. Simple and predictable technique. Better crown/root ratio than surgical crown lengthening.	Rapidity: in just one time it is possible to obtain the extrusion of the desired amount and the correction of endodontic problems, with the possibility to inspect and treat otherwise inaccessible areas without damaging the contiguous elements. Compared to surgical crown lengthening, less bone loss and better maintenance of the interproximal papilla. Compared to orthodontic extrusion: less coronal migration of support tissues and much shorter time of therapy required.
Disadvantages	Long time of treatment required; Worsening oral hygiene and aesthetic problems; High patient compliance required if fiberotomy is performed weekly.	Risk of ankylosis and root resorption due to periodontal ligament trauma; Absence of a universal protocol.
